# Application of spectral CT in the diagnosis of contrast encephalopathy following carotid artery stenting: a case report

**DOI:** 10.1186/s12883-020-01992-x

**Published:** 2020-11-12

**Authors:** Qiuhong Jiang, Liming Shu, Hua Hong

**Affiliations:** 1 Department of Neurology, The First Affiliated Hospital, Sun Yat-sen University; Guangdong Provincial Key Laboratory of Diagnosis and Treatment of Major Neurological Diseases, National Key Clinical Department and Key Discipline of Neurology, No.58 Zhongshan Road 2, Guangzhou, 510080 China; 2 Department of Neurology, The Seventh Affiliated Hospital, Sun Yat-sen University, No. 628, Xinhun Road, Shenzhen, P. R. China

**Keywords:** Contrast encephalopathy, Carotid artery stenting, Spectral CT, Case report

## Abstract

**Background:**

Contrast encephalopathy is a rare complication of carotid artery stenting (CAS). Contrast encephalopathy is a diagnosis of exclusion that often needs to be distinguished from high perfusion syndrome, cerebral haemorrhage, subarachnoid haemorrhage (SAH), cerebral infarction and so on.

**Case presentation:**

In this study, we report on a 70-year-old man who was admitted to the hospital with transient ischaemic attacks presenting paroxysmal weakness of limbs in the previous 2 years. He had severe stenosis of the left internal carotid artery diagnosed by digital subtraction angiography (DSA) and underwent CAS. Two hours after the operation, the patient developed paralysis of the right upper limb, unclear speech, fever and restlessness. Emergency skull computed tomography (CT) showed swelling and a linear high-density area in the left cerebral hemisphere. To clarify the components of this high-density area in the traditional CT, the patient had spectral CT, which made the diagnosis of the leakage of contrast clear. After 1 week of supportive treatment, the patient improved.

**Conclusions:**

Spectral CT can easily distinguish the components of high-density areas on traditional CT, which is haemorrhage, calcification or iodine contrast leakage. Therefore, spectral CT is worth consideration for the differential diagnosis of complications of vascular intervention.

## Background

Common complications after carotid artery stenting (CAS) include hyperperfusion syndrome, plaque detachment and embolism, cerebral haemorrhage, subarachnoid haemorrhage (SAH) or infarction [1]. Contrast encephalopathy is a rare complication of CAS [1, 2]. Its symptoms are similar to stroke, including cortical blindness, speech insufficiency, seizures, restlessness, and focal neurological deficits [3, 4], which makes it difficult to distinguish it from more common complications, such as cerebral haemorrhage (SAH), infarction, and hyperperfusion syndrome [5, 6]. Here, we present the diagnosis of an unusual case of contrast encephalopathy following CAS by using spectral computed tomography (CT).

## Case presentation

**Fig. 1:**
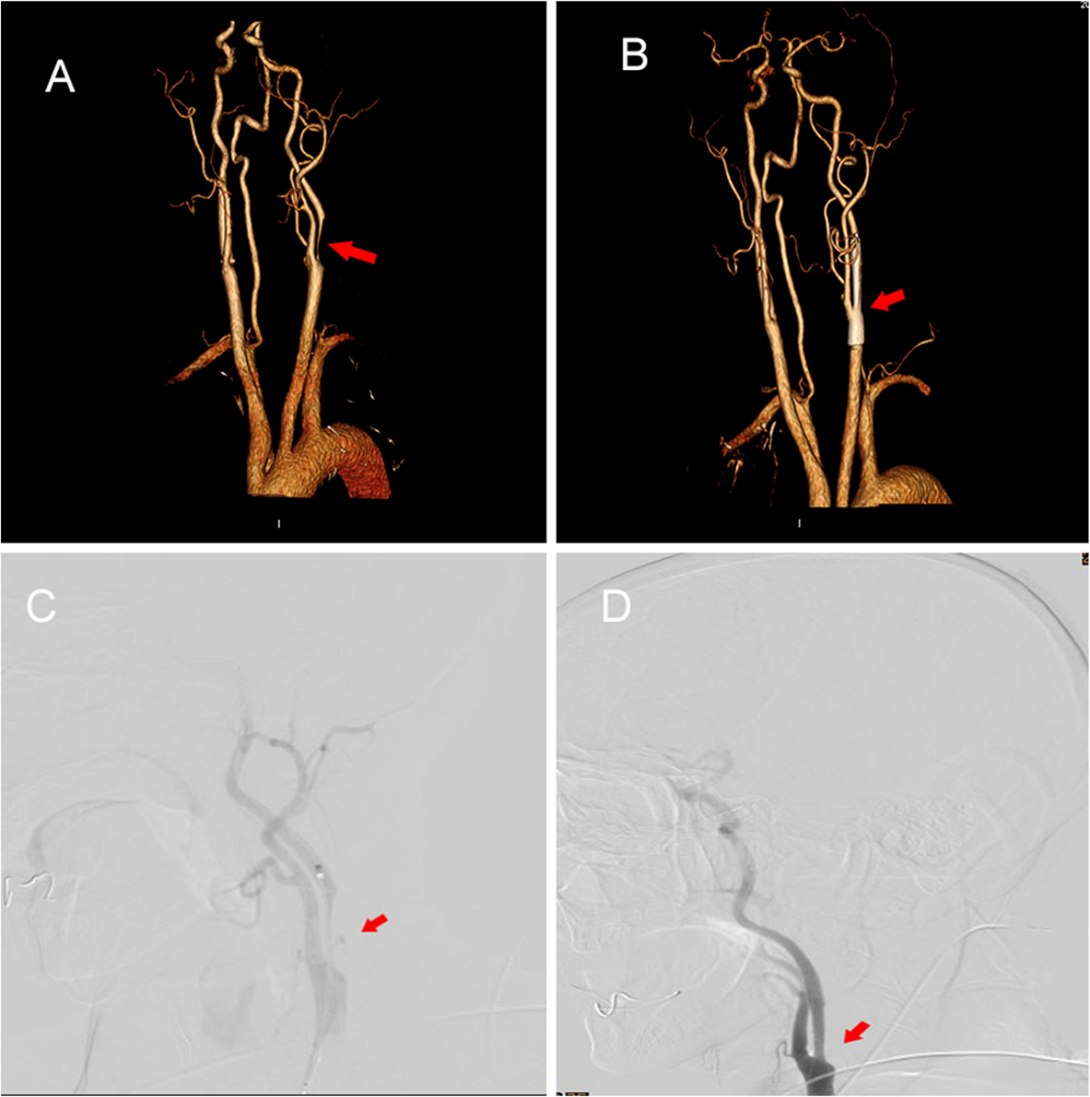
Internal carotid artery of the patient. **a**: Severe left carotid artery stenosis in the neck CTA. **b** Left carotid artery lumen unobstructed after CAS in the neck CTA. **c** Severe left internal carotid artery stenosis in the DSA. **d** Stent implantation lumen unobstructed in the DSA. DSA: digital subtraction angiography

A 70-year-old male was admitted to the hospital on November 7, 2019 with transient ischaemic attacks presenting paroxysmal weakness of the limbs in the previous 2 years. He had a past medical history of diabetes and right inguinal surgery in 2018. On physical examination, his blood pressure was 147/74 mmHg, and a systolic whistle-like noise was heard in his left carotid artery. A neurological examination showed a slightly shallow nasolabial sulcus, and a test for paraplegia showed that the right upper limb was suspected to be positive. His bilateral deep tendon reflexes were (+ ~ ++). Bilateral palmar jaw reflexes were positive, and the right Gordon sign was (±). One-stop CT of the brain was conducted on November 11th: computed tomography angiography (CTA) of the cervical vessels indicated severe stenosis of the left internal carotid artery (Fig. 1a), and CT perfusion imaging (CTP) showed decreased perfusion in the left hemisphere.

**Fig. 2:**
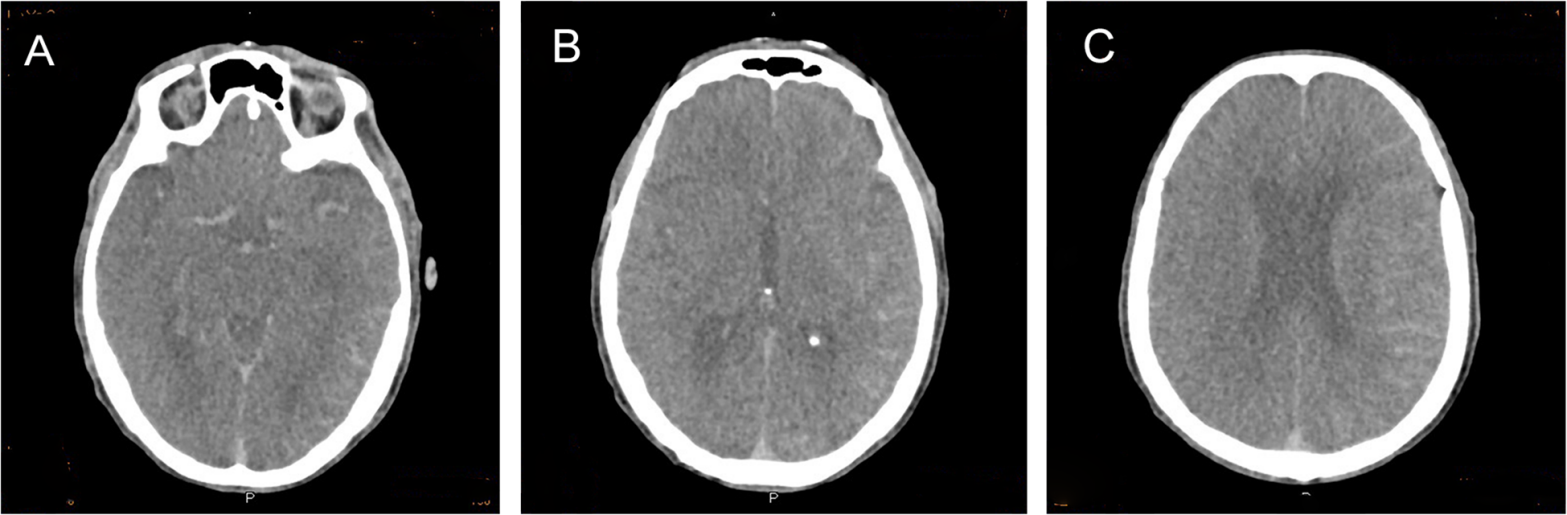
CT scan of the patient’s head at onset; **a**-**c**: Swelling of the left cerebral hemisphere and left linear high-density area

**Fig. 3:**
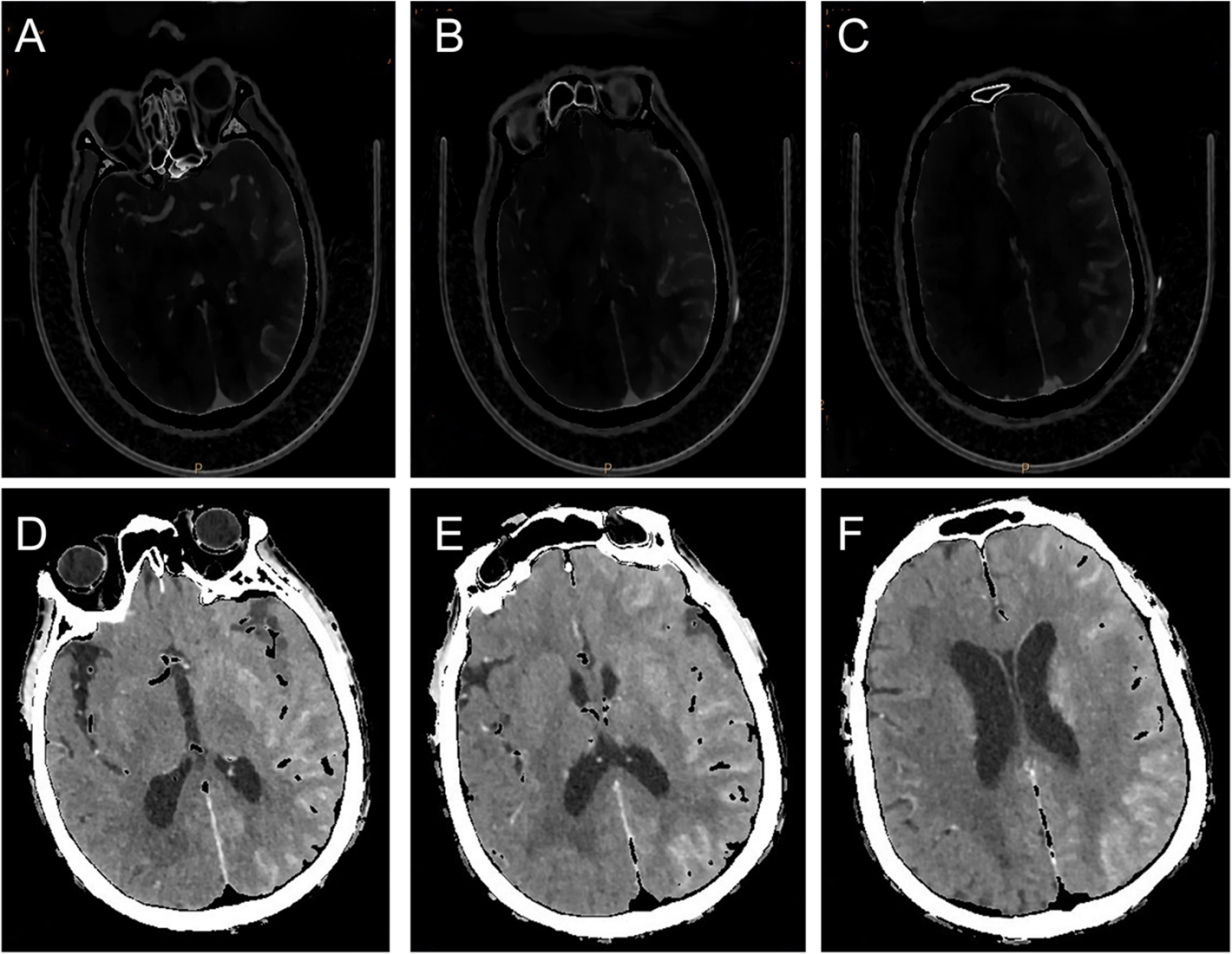
Spectral CT of the patient: linear high-density area in the left hemisphere and the right hemisphere in the image of iodine density; **d-f**: High-density area disappeared and became a blank shadow in the image with the iodine removed

**Fig. 4:**
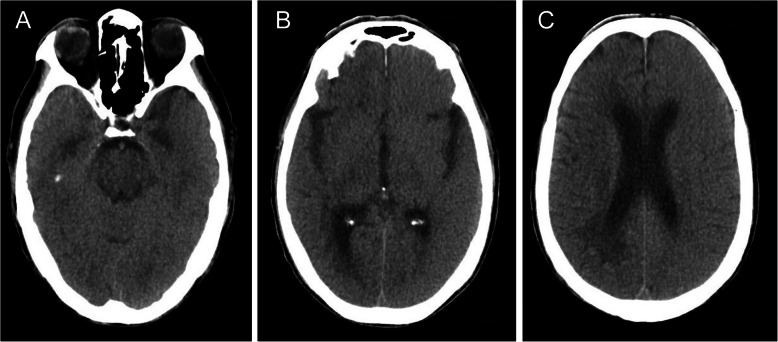
CT scan of the patient’s head after treatment. **a**-**c**: The high-density shadow disappeared, and the oedema was significantly absorbed

After admission, the patient was the patient was treated with nutritive nerve, aspirin antiplate and stable plaque. A week later, the patient underwent CAS. During the operation, severe stenosis was found in the initial segment of the left internal carotid artery (Fig. 1c). After implantation of a self-expanding stent (Wallstent 9 * 40 mm), the left internal carotid artery stent and distal blood flow were smooth (Fig. 1d). The operation was successful, and the patient was safely returned to the ward. Two hours after surgery, the patient developed weakness in the right hand and poor speech. On physical examination, his heart rate was 73, and his blood pressure was 110/62 mmHg. Right upper limb muscle strength was grade 1 to 2. He immediately underwent another one-stop CT examination, which showed swelling of the left frontal parietal lobe and a linear high-density area in the cortex (Fig. 2a-c). A stent shadow could be seen in the left internal carotid artery, and the lumen was unobstructed. CTP showed no high perfusion image and was basically the same as the previous scan. To determine the cause of the left hemisphere lesions, the Philips IQon spectral CT (Philips Healthcare, Cleveland, OH, USA) was performed at the same time. The iodine density image indicated a scattered high-density area in the left hemisphere, which was also somewhat apparent in the right hemisphere, while the scattered high-density area disappeared and became a blank shadow on the image once the iodine had been removed (Fig. 3a-e). He was diagnosed with contrast encephalopathy. He was immediately treated with dexamethasone, mannitol and albumin to induce dehydration and reduce cranial pressure. Six hours after surgery, he presented as restless, he wanted to sit up and get out of bed, and he had a tendency to attack others. He was immediately given 10 mg of diazepam. At night, he developed fever, with a maximum temperature of 39 degrees Celsius. He was treated with ice compress, aminobarbital, anti-infectives, and midazolam for sedation. After 1 week of supportive treatment, the patient improved. The muscle strength of the right upper limb was 5 -, the speech was fluent, and there was no restlessness or other discomfort. Reexamination of head CT showed that the swelling of the left hemisphere was less than before, and the linear high-density area in the cortex had disappeared (Fig. 4a-c). One month after discharge, the follow-up by telephone revealed that the patient had completely recovered without recurrence.

## Discussion and conclusion

CAS is widely used in the treatment of atherosclerotic diseases. It is rare that the use of contrast in these operations causes contrast encephalopathy. Symptoms usually appear 2–12 h after contrast administration. Most patients fully recover within 2–3 days, but a small number of patients die from irreversible cerebral oedema and persistent neurological deficits [4, 7, 8]. It is important to accurately distinguish these diseases because of the similarity to the complications of haemorrhage, SAH embolism and infarction after CAS [9].

Two hours after CAS, our patient presented with right upper limb paralysis, accompanied by restlessness, fever and other symptoms. Emergency skull CT showed swelling and a linear high-density area in the left cerebral hemisphere. CTP showed no obvious hyper-perfusion signs. The artery and vessel cavity of the stent was unobstructed, and no thrombosis was found. Therefore, it was difficult to distinguish whether the left linear high-density area was cerebral haemorrhage or was a leakage of contrast medium. To clarify the components of the left linear high-density area in the traditional CT, the patient was examined with spectral CT. The image of iodine density also indicated a left linear high-density area—moreover, a high-density area was also observed in the right hemisphere that was not found in the traditional CT—but the high-density areas disappeared and became a blank shadow in the image with the iodine removed, which made the diagnosis of the leakage of contrast clear.

Spectral CT is a CT imaging method based on rapidly switching between high-energy and low-energy data, which can be used to generate material decomposition images and monochromatic spectrum images with energy levels of 40–140 Kev [10]. It includes two technologies: material separation and energy spectrum curve. Energy spectrum curves are generated by plotting the CT attenuation value of a material for every monochromatic energy from 40 to 140 Kev, which helps to characterize specific tissue types because the curves are based on the known mean attenuation characteristics of given material^s^ [11]. In the process of material separation, two different materials can be arbitrarily selected by complex material decomposition algorithms. Materials with low and high densities, such as water and iodine, are often used as the basis pair for medical diagnostic imaging. Then, various material decomposition images can be generated with this process [12]. Combining the material separation technology and energy spectrum curve technology of spectral CT, we can distinguish whether the high-density area observed in the traditional CT was due to calcification, haemorrhage or contrast leakage [11]. Spectral CT’s single-energy imaging technology can not only improve the signal-to-noise ratio and make the image clearer but also eliminate hardening artefacts [13]. In our patient, we used the material separation technology of spectral CT to identify the components of the high-density shadows in the brain that were first observed after CAS. This approach provides a new strategy for the identification of the components of high-density areas observed in traditional CT.

At present, the mechanism of contrast encephalopathy is still unknown [14]. Generally, macromolecules such as iodine contrast cannot enter the brain through the blood-brain barrier (BBB). When the integrity of the BBB is destroyed, the iodine contrast can enter the central nervous system, which leads to direct chemical toxicity. Meanwhile, the permeability to the iodine contrast will pull liquid into the brain, leading to brain oedema. One of the theories regarding the destruction of the BBB by iodine contrast is that the permeability to hypertonic or ionic contrast can dehydrate the vascular endothelial cells, cause contraction of endothelial cells, expand the tight connections, and make the macromolecular substances (such as iodine contrast) in the blood vessels flow out of the blood vessels [15, 16]. In regard to the limitations of spectral CT, it may be that performing multiple spectral reconstructions can be expected to increase the reconstruction time and expense of archiving additional images. In addition, reviewing additional images on dedicated thin client servers may increase radiologists’ interpretation time [17].

Overall, contrast encephalopathy is a rare complication in CAS. Moreover, contrast encephalopathy is a disease diagnosed by exclusion because of its similarity to intracerebral haemorrhage, SAH and cerebral infarction. The advantages of spectral CT compared with traditional CT are that it can distinguish whether the components of high-density areas are a result of haemorrhage, calcification or iodine contrast leakage, which makes it easier and more accurate to diagnose intracranial lesions. Therefore, spectral CT is worth recommending in the differential diagnosis of complications of vascular intervention.

## Data Availability

The datasets supporting the conclusions of this article are included within the article.

## Abbreviations

CAS: Carotid artery stenting
SAH: Subarachnoid haemorrhage
DSA: Digital subtraction angiography
CTA: Computed tomography angiography
CTP: CT perfusion imaging
BBB: Blood-brain barrier
